# Immune response against rickettsiae: lessons from murine infection models

**DOI:** 10.1007/s00430-017-0514-1

**Published:** 2017-08-02

**Authors:** Anke Osterloh

**Affiliations:** Bernhard Nocht Institute for Tropical Medicine, Hamburg, Germany

**Keywords:** Rickettsiae, Immunity, Immunopathology

## Abstract

Rickettsiae are small intracellular bacteria that can cause life-threatening febrile diseases. Rickettsioses occur worldwide with increasing incidence. Therefore, a vaccine is highly desired. A prerequisite for the development of a vaccine is the knowledge of the immune response against these bacteria, in particular protective immunity. In recent years murine models of rickettsial infections have been established, and the study of immune response against rickettsiae in mice provided many new insights into protective and pathological immune reactions. This review summarizes the current knowledge about immune mechanisms in protection and pathology in rickettsial infections.

## Introduction

### Rickettsioses

Rickettsioses are emerging and reemerging febrile diseases that are caused by small obligate intracellular bacteria of the family of *Rickettsiaceae*. *Rickettsiaceae* are currently divided into two genera, *Rickettsia* and *Orientia*, and a third genus (*Occidentia*) has recently been proposed [1]. While *Orientia* (*O.*) *tsutsugamushi* is the only member of the genus *Orientia* and the causative agent of scrub typhus, the genus *Rickettsia* is further dIvided into four major groups. The vast majority of approximately 20 rickettsial species belongs to the spotted fever group (SFG), e.g. *Rickettsia* (*R*). *rickettsii* and *R. conorii*, the causative agents of Rocky Mountain spotted fever (RMSF) and Mediterranean spotted fever (MSF). *R. prowazekii* and *R. typhi* are the members of the typhus group (TG) of rickettsiae. These bacteria cause epidemic and endemic typhus, respectively. *R. felis*, *R. akari* and *R. australis* belong to the transitional group of pathogenic rickettsiae while members of the ancestral group (*R. bellii*, *R. canadensis*) are non-pathogenic [2–4]. Rickettsiae are transmitted to humans by arthropod vectors and enter the body during the blood meal of the arthropod. Table 1 provides an overview of the genera *Rickettsia* and *Orientia*, vectors and natural reservoirs, the disease that is induced by different rickettsial species and their distribution (Table 1).

**Table 1:** The family of *Rickettsiaceae*

Genus	Group	Species	Vector	Reservoir	Disease/symptoms	Distribution
*Rickettsia*	SFG	*R. rickettsii*	Tick	Rodents	Rocky Mountain SF	North, Central and South America
		*R. conorii*	Tick	Rodents	Mediterranean SF	Europe, Asia, Africa
		*R. honei*	Tick	Rodents	Flinder´s island SF	Australia, Thailand
		*R. japonica*	Tick	Rodents	Japanese SF/oriental SF	Japan
		*R. sibirica*	Tick	Rodents	North Asian tick typhus	Russia, China, Mongolia, Europe
		*R. africae*	Tick	Rodents	African tick bite fever	Sub-Saharan Africa, Caribbean
		*R. slovaca*	Tick	Rodents	Necrosis, erythema	Europe
		*R. helvetica*	Tick	Rodents	Aneruptive fever	Africa, Europe, Asia
		*R. parkeri*	Tick	Rodents	Mild spotted fever	US, Brazil, Uruguay
	TG	*R. prowazekii*	Louse	Human, flying squirrel	Epidemic typhus	Worldwide
		*R. typhi*	Flea	Rodents, cats, dogs	Endemic typhus	Worldwide
	Transitional	*R. felis*	Flea	Rodents, cats, opossums	Cat flea typhus	Worldwide
		*R. akari*	Mite	Rodents	Rickettsialpox	Worldwide
		*R. australis*	Tick	Rodents	Queensland tick typhus	Australia, Tasmania
	Ancestral	*R. bellii*	Tick			
		*R. canadensis*	Tick			
*Orientia*		*O. tsutsugamushi*	Mite	Rodents	Scrub typhus	Indian subcontinent, Asia, Australia

The family of *Rickettsiceae* consists of two genera: *Rickettsia* and *Orientia*. *O. tsutsugamushi* is the only member of the genus *Orientia*. The genus *Rickettsia* is subdivided into four groups: SFG (spotted fever group), TG (typhus group), transitional and ancestral rickettsiae. The vast majority of rickettsiae belongs to the SFG. *SF* spotted fever (adapted and modified from [5])

**Fig. 1:**
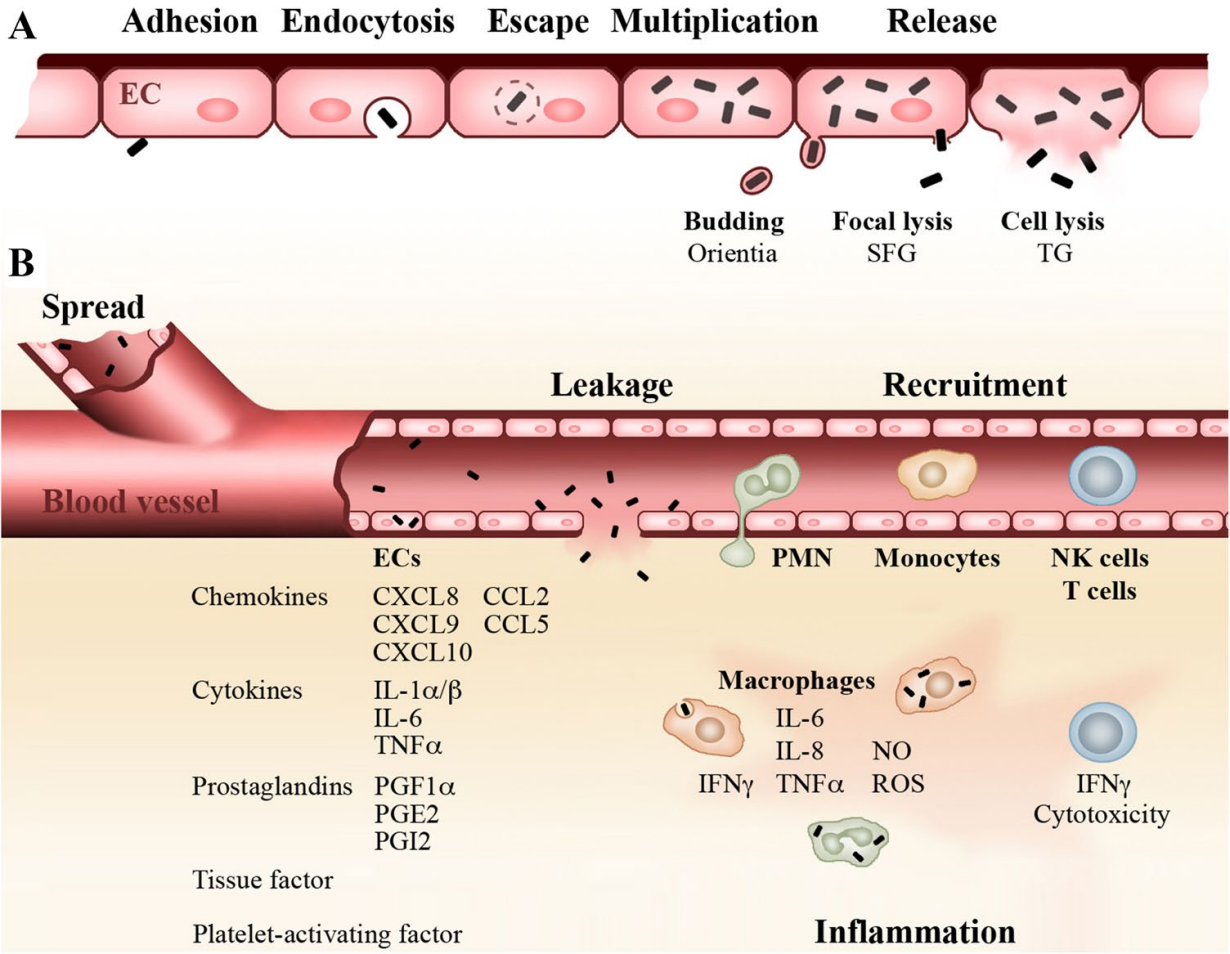
Rickettsiae replicate in ECs and induce local inflammatory reactions. Rickettsiae enter ECs by endocytosis and rapidly escape from the endosome. The bacteria replicate free in the cytosol and are released by different mechanisms. SFG rickettsiae are capable to induce focal lysis while TG rickettsiae replicate in the cell until burst. *O. tsutsugamushi* induces a kind of budding (**a**). Rickettsiae infect adjacent ECs, further spread via the blood stream and enter the tissue via local lesions. Infected ECs release a series of chemokines, cytokines, prostaglandins and other factors. Chemokines attract neutrophils, monocytes/MΦ, NK cells and T cells from the periphery into the affected tissue. MΦ and neutrophils both of which also get infected with rickettsiae release NO and ROS which is important for killing of ingested bacteria. The production of NO is supported by IFNγ which is provided by infiltrating NK cells and T cells. MΦ and infiltrating NK and T cells further produce inflammatory cytokines such as TNFα that contribute to local inflammation (**b**)

Rickettsiae infect endothelial cells (ECs) that coat the inner wall of the blood vessels. These cells are considered the dominant target cells of rickettsiae [3, 6]. The bacteria are taken up by endocytosis and rapidly escape from the endosome by endosomal lysis [7–11]. Rickettsiae then replicate free in the cytosol until release by different mechanisms. SFG Rickettsiae are believed to induce targeted focal membrane lysis for exit, allowing cell-to-cell spread without destruction of the host cell [12, 13]. TG rickettsiae that miss an appropriate actin tail for directed movement [14, 15] grow in the cell until lysis or burst [13], and *O*. *tsutsugamushi* exits the cell by a budding-like process [16] (Fig. 1a). Free bacteria are then capable to infect adjacent cells. Local lesions of the blood vessels can lead to edema and thromboses. ECs in addition to tissue macrophages (MΦ) release a series of cytokines, chemokines, and other mediators that lead to the recruitment and activation of immune cells that further promote local inflammatory responses. Figure 1b provides an overview on local reactions in response to rickettsiae. Due to these reactions, the location of entry of several rickettsiae (SFG Rickettsiae, *O. tsutsugamushi* and *R. prowazekii*) is usually associated with an eschar. The bacteria further spread via the blood stream and systemically distribute in the body during the course of disease, inducing inflammatory responses in the organs and in the skin where these reactions become visible as a characteristic spotted skin rash in 60–70% of the patients. Rickettsiae can enter nearly all tissues and organs. Apart from ECs they also infect monocytes/MΦ [3, 6, 17] and non-immune cells including hepatocytes [18, 19], smooth muscle cells [20], neurons [21] and fibroblasts that are commonly used for in vitro culture of rickettsiae [22–25]. Humans develop disease upon rickettsial infections within 10–14 days after inoculation. Common symptoms include fever, headache, myalgia, muscle aches, cough and rash. Severe complications such as pneumonia, heart and liver damage, nephritis, encephalitis or meningitis can occur and lead to a fatal outcome. The highest lethality (20–30%) is observed in the infection with *R. prowazekii* [26–28].

Rickettsial infections are treated with antibiotics. A vaccine, however, is not available but highly desired for several reasons: (1) Rickettsial diseases are emerging worldwide with increasing incidence. (2) There is the risk of the development of antibiotic resistance. (3) The bacteria can persist despite antibiotic treatment. This is known for *R. prowazekii* [29], *O. tsutsugamushi* [30] *R. rickettsii* [31, 32] and quite likely for *R. typhi* [33]. *R. prowazekii* can be reactivated decades after primary infection and cause the so-called Brill-Zinsser disease [34–37]. Reactivation of persisting bacteria and recurrent disease may also occur in the infection with other rickettsiae. (4) Finally, rickettsiae are considered potential bioweapons.

### Murine models of rickettsial infections

Vaccine development requires the understanding of immune mechanisms that are involved in protection and pathology. In recent years, immune response in rickettsial infections has been intensively studied in murine models of infection. Susceptible mouse strains reflect human disease in many aspects, including the development of vasculitis, pneumonia, hepatitis, meningoencephalitis and splenomegaly. C3H/HeN mice are susceptible to a broader range of rickettsiae while BALB/c and C57BL/6 mice that are commonly used to study immunity against infectious pathogens are resistant to most rickettsial infections. However, knockout mice from these strains that lack immune components provided much insight into immunity against rickettsial infections as described later. Table 2 provides an overview of murine models of rickettsial infections and the outcome of disease.

**Table 2:** Murine models of rickettsial infections

Strain	Species	Route	Suscept.	Pathology	Persistence	References
**BALB/c**						
Wild-type	*R. australis*	i.v.	**++**	Vasculitis, pneumonia, hepatitis		[38]
	*R. akari*	i.p.	−			[39]
	*R. typhi*	s.c., i.v.	−	Pneumonia, hepatitis (mild)	yes	[33, 40]+Osterloh unpubl.
	*O. tsutsugamushi*	s.c., i.p.	−	Pneumonia, hepatits, myocarditis, meningoencephalitis	yes	[41–43]
CB17	*R. conorii*	i.v.	−	Mild hepatitis		[44]
CB17 SCID	*R. conorii*	i.v.	−	Mild hepatitis		[44]
	*R. typhi*	s.c.	**++++**	Strong hepatitis, splenomegaly, systemic inflammation (IFNγ)		[40, 45]
RAG2^−/−^	*R. typhi*	s.c.	**++++**	Strong hepatitis, splenomegaly, systemic inflammation (IFNγ)		[45]
IFNγ^−/−^	*R. typhi*	s.c.	−			[45]
Perforin^−/−^	*R. typhi*	s.c.	−			[45]
**C57BL/6**						
Wild-type	*R. australis*	i.v.	**+**			[46]
	*R. akari*	i.p.	−			[39]
	*R. conorii*	i.v.	−			[46]
	*R. typhi*	s.c., i.v.	−	Mild hepatitis, pneumonia	yes	[33, 47]+Osterloh unpubl.
	*O. tsutsugamushi*	i.p.	−			[43]
MHCI^−/−^	*R. australis*	i.v.	**+++**	Encephalitis		[46]
	*R. typhi*	s.c.	−			[47]
MHCII^−/−^	*R. typhi*	s.c.	−			[47]
RAG2^−/−^	*R. conorii*	i.v.	− (?)	Survival for at least 21 days, mild hepatitis		[44, 46]
RAG1^−/−^	*R. typhi*	s.c., i.v.	**++++**	Survival for >3 months, lethal CNS inflammation	yes	[33]
RAG2^−/−^γc^−/−^	*R. conorii*	i.v.	**++**	Enhanced hepatitis		[44]
RAG2^−/−^Perforin^−/−^	*R. conorii*	i.v.	**+**			[44]
Perforin^−/−^	*R. australis*	i.v.	**+++**			[44, 46]
IFNγ^−^/^−^	*R. australis*	i.v.	**++**			[46]
NLRP3^−/−^	*R. australis*	i.v.	**++**			[48]
**C3H/HeN**						
Wild-type	*R. akari*	i.p.	−			[39]
	*R. conorii*	i.v.	**++**	Meningoencephalitis, pneumonia, hepatitis		[18, 44]
	*R. typhi*	i.v.	**+**	Encephalitis, pneumonia		[49]
	*O. tsutsugamushi*	i.p.	**++**			[43]
	*R. parkeri*	i.v.	−			[50]
C3H/HeJ	*R. australis*	i.p.	−			[51]
	*R. akari*	i.p.	**+**			[39]
	*R. conorii*	i.v.	**+++**			[52]
	*R. rickettsii*	i.p	−			[51]
	*R. sibirica*	i.p.	**++**			[51]
	*R. parkeri*	i.v	**+++**	Splenomegaly		[50]
C3H/HeN SCID	*R. conorii*	i.v..	**++++**			[53]

In recent years immune response against rickettsiae has been intensively studied in murine models of infection. BALB/c and C57BL/6 mice that are commonly used for the study of immunity against infectious pathogens are resistant against most rickettsiae while C3H/HeN mice are susceptible to a broader range of rickettsiae. Except for C3H/HeJ mice that are deficient in the expression of a functional TLR4 receptor, knockout mice on the C3H background are not available. Much knowledge about protective immunity against rickettsial infections has been gained from the comparison of rickettsial infections in BALB/c and C57BL/6 wild-type mice and mice of these backgrounds that either lack components of adaptive immunity or effector molecules such as IFNγ and Perforin. Overall, the enhanced susceptibility of BALB/c or C57BL/6 mice that either lack T and B cells (CB17 SCID, RAG2^−/−^ and RAG1^−/−^ mice), CD8^+^ T cells (MHCI^−/−^) or CD4^+^ T cells (MHCII^−/−^) to rickettsial infections demonstrates the importance of adaptive immunity, especially of T cells, in defense against these pathogens

*Suscept.* susceptibility; *i.v.* intravenous; *i.p.* intraperitoneal; *s.c.* subcutaneous

## Immune response against rickettsiae

### Response of non-immune target cells to rickettsial infections

Although ECs are non-immune cells, once infected with rickettsiae they produce a series of mediators that contribute to the recruitment and activation of immune cells. Rickettsia-infected ECs release several chemokines that induce the recruitment and infiltration of immune cells from the periphery. These include CXCL8/IL-8, CCL2/MCP-1 (monocyte chemoattractant protein 1) [54, 55], CXCL9/MIG (monokine-induced by gamma interferon), CXCL10/IP10 (interferon-gamma inducible protein 10) [56, 57] and CCL5/RANTES [57]. CXCL8/IL-8 predominantly recruits neutrophils to the site of infection via CXCR1 and 2 [58–60]. It activates exocytosis and the production of bactericidal reactive oxygen species (ROS) in neutrophils [58] and in this way acts pro-inflammatory. CXCL8/IL-8 further has anti-apoptotic effects on ECs, promotes endothelial proliferation and angiogenesis [61]. CCL2/MCP-1 and CCL5/RANTES are involved in the recruitment of monocytes and other leukocytes that express CCR1, 2, 3 or 5 [62, 63] while CXCL9 and CXCL10 attract activated NK and T cells, predominantly CD4^+^ T_H_1 cells and CD8^+^ cytotoxic T cells, via CXCR3 [64]. ECs further upregulate the expression of adhesion molecules such as E-selectin which facilitates neutrophil adhesion [65] and ICAM-1 [66], promoting the adhesion and transmigration of activated leukocytes [67].

ECs activate infiltrating immune cells by the release of inflammatory cytokines including IL-1β, IL-6 and TNFα [57, 68]. IL-1β activates similar responses in innate immune cells as the recognition of pathogens via Toll-like receptors (TLR) [69]. IL-6 drives the differentiation of infiltrating monocytes to MΦ, enhances the production of CCL2/MCP-1, CCL8/MCP-2, CXCL5/ENA-78 and CXCL6/GCP-2 in tissue cells, the expression of ICAM-1, VCAM-1 and E-selectin on ECs as well as L-selectin on lymphocytes [70]. In this way IL-6 enhances leukocyte transmigration. TNFα has diverse effects and can locally activate MΦ for bacterial killing [71].

Finally, rickettsia-infected ECs produce prostaglandins including the vasoactive prostaglandins PGF1α, PGI2 and PGE2 [72, 73], PAF (platelet-activating factor), a mediator of platelet aggregation, degranulation and vascular permeability [74], and TF (tissue factor) [75] which is induced by IL-1β in an autocrine manner [68]. TF is an integral membrane protein that interacts with plasma factor VII when expressed on damaged ECs and initiates blood coagulation [76]. The transcription factor NF-κB that gets activated in rickettsia-infected ECs [77] has been demonstrated to be involved in many of these events including the expression of TF [78], CXCL8, und CCL2 [54]. In addition, NF-κB acts as an anti-apoptotic factor for rickettsia-infected ECs [79].

### Role of innate immune cells in protection and pathology

#### Phagocytes

Apart from a series of non-immune cells also cells of the innate immune system, especially phagocytes such as MΦ, that are recruited to the location of infection can get infected by rickettsiae. Various rickettsiae infect primary MΦ as well as MΦ cell lines in vitro and replicate within these cells. In fact, some rickettsiae seem to predominantly infect MΦ rather than ECs. This is postulated for *R. akari* [80] and may be also true for *O. tsutsugamushi*, *R. conorii* and *R. typhi*. *O. tsutsugamushi* is found predominantly in MΦ in the tissues of infected BALB/c mice [41], and both *O. tsutsugamushi* and *R. conorii* are detectable in hepatic MΦ, most likely Kupffer cells, in the liver of infected mice [18, 81]. *R. typhi* is exclusively detectable in MΦ, but not in ECs, in CB17 SCID and C57BL/6 RAG1^−/−^ mice with advanced infection [33, 40].

The activation of MΦ upon infection depends on the rickettsial species. *R. australis* induces the release of IL-1β and IL-18 in bone marrow-derived MΦ from BALB/c mice [48]. *R. australis*-infected C57BL/6 and BALB/c mice show enhanced serum levels of IL-1β, IL-6, IL-12p40 and TNFα [38, 82]. Correspondingly, serum levels of IL-6 and IL-12 are increased in C3H/HeN mice upon *R. conorii* infection [52]. These cytokines are typically produced by MΦ. *R. australis* further activates Caspase-1 as well as the inflammasome in bone marrow-derived MΦ from BALB/c mice [48]. The latter seems to play a role in the control of this pathogen because *R. australis*-infected C57BL/6 NLRP3^−/−^ mice that are deficient in the formation of the inflammasome show enhanced bacterial burden in the spleen, although not in other organs [48]. *R. akari* induces the activation of NF-κB and the expression of IL-1β, IL-6 and TNFα in P338D1 MΦ and peritoneal MΦ from C3H/HeN mice [17]. The same has been observed upon the infection of peritoneal MΦ from C3H/HeN mice with *R. typhi* that additionally induced the release of TGFβ [17]. In other studies, however, bone marrow-derived MΦ from BALB/c and C57BL/6 mice neither produced cytokines nor nitric oxide (NO) upon infection with *R. typhi,* but exclusively upregulated MHCI and CD80 on the cell surface [40, 47]. Although there may be differences in the reactivity of MΦ from different mouse strains and also between peritoneal and bone marrow-derived MΦ, these observations argue against classical activation of MΦ by *R. typhi* which is usually mediated by pathogen recognition via pathogen recognition receptors such as TLR.

These receptors typically activate the transcription factor NF-κB which induces the release of several inflammatory cytokines (IL-6, IL-12, TNFα), the expression of inducible nitric oxide synthase (iNOS) which produces NO, the upregulation of MHC class I and II and costimulatory CD80 and CD86 molecules [83]. TLR4 and TLR2 seem to be engaged in the activation of innate immune response. For example, *R. akari* activates NF-κB in reporter cell lines via TLR4 and also TLR2 [84]. TLR4 has also been implicated in protection against rickettsiae in vivo. C3H/HeJ mice that lack a functional TLR4 show enhanced susceptibility to *R. conorii* and develop overwhelming bacterial burden and fatal disease when infected with a normally sublethal dose [52]. Similar observations were also made for the infection of C3H/HeJ mice with *R. parkeri* [50]. An important role of TLR in defense against rickettsiae is further supported by the finding that C57BL/6 MyD88^−/−^ mice that lack the conserved MyD88 signaling pathway which is common to TLRs [83] succumb to the infection with *R. conorii* as well as with *R. australis* [82]. In addition, *R. australis*-infected C57BL/6 MyD88^−/−^ show reduced serum levels of IL-6, IL-12p40, TNFα and also IFNγ compared to wild-type mice [82] which is also true for *R. conorii*-infected C3H/HeJ mice that produce reduced serum levels of IL-6 and IL-12 and show enhanced numbers of regulatory T cells compared to C3H/HeN wild-type mice [52]. These findings indicate missing innate immune activation by these rickettsial species in the absence of TLR4 which is essential for the initiation of protective adaptive immunity.

The role of TLR in protection against *O. tsutsugamushi* and also TG rickettsiae, however, is not clear. In the infection with *O. tsutsugamushi* TLR2, but not TLR4, is required for the release of TNFα and IL-6 by dendritic cells (DCs) upon pathogen contact [85]. C57BL/6 TLR2^−/−^ mice, however, are more resistant to lethal infection with *O. tsutsugamushi* compared to C57BL/6 wild-type mice [85]. Although C57BL/6 TLR2^−/−^ mice develop a higher bacterial burden compared to C57BL/6 wild-type mice in the infection with *O. tsutsugamushi*, C57BL/6 TLR2^−/−^ mice show milder symptoms of disease and enhanced survival rates [85]. These findings show that the activation of innate immunity via TLR2 although being involved in bacterial elimination in this infection clearly has pathological effects that are largely responsible for disease.

Immunopathology mediated by innate immune mechanisms is also responsible for the lethal outcome of the infection of CB17 SCID mice and C57BL/6 RAG1^−/−^ mice with *R. typhi*. CB17 SCID mice succumb to the infection with *R. typhi* within 3 weeks, develop splenomegaly due to the massive expansion of MΦ and neutrophils, and severe liver necrosis [40]. Liver damage in these mice is exclusively mediated by the action of infiltrating neutrophils [40]. Neutrophils and MΦ express iNOS, release reactive oxygen species (ROS) and TNFα in the infection with *R. typhi* [40], and death of the animals can be ascribed to overwhelming systemic release of inflammatory cytokines [40]. Systemic inflammation is clearly dominated by the production of IFNγ for which NK cells as well as MΦ are the major source in *R. typhi*-infected CB17 SCID mice [40]. In contrast to CB17 SCID mice, C57BL/6 RAG1^−/−^ mice can control *R. typhi* for 3–4 months before the bacteria start to grow predominantly in the brain [33]. This leads to fatal inflammation of the central nervous system (CNS) which is associated with the infiltration of activated MΦ from the periphery and the expansion and activation of microglia. In the CNS of these mice, *R. typhi* is almost exclusively found in infiltrating MΦ [33], and the same is also true for *R. typhi*-infected CB17 SCID mice where the bacteria reside in MΦ and neutrophils [40]. How these cells get activated in the infection of CB17 SCID and C57BL/6 RAG1^−/−^ mice, however, is unclear. The majority of activated MΦ and neutrophils that express iNOS in *R. typhi*-infected mice does not harbor the bacteria [40]. Together with the finding that *R. typhi* does not activate bone marrow-derived MΦ from BALB/c and C57BL/6 mice in vitro in a classical manner [40, 47], a role of TLR in the activation of innate immune response by TG rickettsiae such as *R. typhi* is questioned. It has been suggested that activation of MΦ in the infection with *R. typhi* in vivo is mediated by indirect mechanisms such as the release of endogenous danger signals by damaged cells rather than direct recognition of the bacteria [40].

Although MΦ react to the infection with rickettsiae, they are incapable of killing the ingested bacteria without help and the bacteria replicate within these cells in vitro. In addition, some rickettsiae such as *O. tsutsugamushi*, *R. typhi*, *R. conorii* and *R. parkeri* seem to predominantly infect MΦ in vivo [18, 33, 40, 80, 81]. Therefore, it is speculated that some rickettsiae may use MΦ as a vehicle for transport through the body. Such “shuttle” service is discussed for *O. tsutsugamushi* [86] as well as for *R. typhi* [33].

Bacterial killing by MΦ and also other cells can be induced by cytokines. Important cytokines that can activate the bactericidal activity of MΦ are IFNγ and TNFα. IFNγ induces the expression of iNOS and subsequent release of NO by MΦ and also endothelial cells [87–89]. TNFα also induces the expression of iNOS in MΦ [87] and synergizes with IFNγ in this effect [71]. IFNγ has been shown to inhibit the growth of *R. prowazekii* in murine and human fibroblasts [90], and IFNγ as well as TNFα inhibit the growth of *R. typhi* in murine MΦ in vitro [45]. IFNγ and TNFα as well as IL-1β and RANTES also activate bacterial killing of *R. conorii* in human THP1 MΦ, AKN-1 hepatocytes and HUVEC ECs via NO-dependent mechanisms [91].

### Natural killer (NK) cells

Natural killer cells are innate cytotoxic cells that are capable of direct killing of infected target cells and have been implicated in early innate defense against rickettsial infections. Enhanced NK cell activity has been reported early in infection on days 2–6 in *R. conorii*-infected C3H/HeN mice [92]. At the same time C3H/HeN mice show enhanced serum levels of IFNγ early in the infection with *R. conorii* which could be ascribed to NK cells. IFNγ serum levels were significantly reduced upon NK cell depletion by antibody, and the mice showed enhanced susceptibility to the infection with *R. conorii* [92]. Furthermore, C57BL/6 mice that are resistant to rickettsial infections show enhanced NK cell activity and IFNγ production early in the infection with *R. conorii* compared to susceptible C3H/HeN mice [44]. Moreover, C57BL/6 RAG2^−/−^γc^−/−^ mice that lack not only T and B cells but also NK cells [93] produce reduced serum levels of IFNγ and develop enhanced bacterial burden and liver pathology in the infection with *R. conorii* compared to NK cell-competent C57BL/6 RAG2^−/−^ mice [44]. Similarly, NK cell-depleted CB17 SCID mice show enhanced susceptibility to the infection with *R. conorii* compared to CB17 mice [44]. These findings indicate an important role of NK cells and NK cell-derived IFNγ in early rickettsial defense which seems to be mainly mediated via the production of IFNγ rather than the cytotoxic activity because *R. conorii*-infected C57BL/6 RAG2^−/−^Perforin^−/−^ mice produce higher levels of IFNγ and have lower bacterial burden compared to C57BL/6 RAG1^−/−^ mice [44]. *R. conorii*-infected C57BL/6 RAG2^−/−^Perforin^−/−^ mice, however, develop enhanced liver pathology despite reduced bacterial burden [44], indicating immunopathology rather than direct cellular damage by the bacteria.

Enhanced NK cell activity has also been reported early in the infection with *R. typhi* on day 2 in C57BL/6 mice [92], and NK cells have been implicated in early defense against *R. typhi* in mice [94]. For example, enhanced bacterial burden was observed in *R. typhi*-infected C57BL/6 mice upon NK cell depletion [92]. The contribution of NK cells to the control of *R. typhi*, however, is not that clear. NK cells did not significantly expand in the initial phase of infection (30 days) in *R. typhi*-infected C57BL/6 RAG1^−/−^ mice, and the animals did not show any signs of disease upon depletion of NK cells during this time [33]. *R. typhi*-infected C57BL/6 RAG1^−/−^ mice further developed neurological disease with similar kinetics as control animals 3 months post infection [33]. In addition, although enhanced serum levels of IFNγ were observed in *R. typhi*-infected C57BL/6 RAG1^−/−^ mice on day 3 post infection, IFNγ release was unaltered upon NK cell depletion [33], demonstrating that early IFNγ in these mice does not predominantly derive from NK cells but other cells, e.g. MΦ and innate lymphoid cells. Only moderate increase of NK cells was also observed in *R. typhi*-infected CB17 SCID mice, and the high amounts of IFNγ that are detectable in these mice equally derive from NK cells and MΦ [40]. Despite the activation of NK cells and MΦ in the infection, CB17 SCID mice are incapable to control the bacteria and develop high bacterial burden, predominantly in the spleen [40]. Together with the findings in *R. typhi*-infected C57BL/6 RAG1^−/−^ mice, these observations question an important role of NK cells in defense against TG rickettsiae and demonstrate that protective cytokines such as IFNγ and TNFα have to be locally provided by cells of the adaptive immune system to act protective.

### Adaptive immune response to rickettsial infections

#### B cells and the role of antibodies in protection against rickettsiae

Adaptive immunity is essential for protection against rickettsiae. This is clearly reflected by the observation that T- and B cell-deficient mice are highly susceptible to rickettsial infections [33, 40, 53]. B cells can contribute to protection by the production of antibodies. Here, it seems that antibodies that are produced in the absence of CD4^+^ T cell help as it is the case in the early phase of infection are less protective than antibodies generated with T cell help. This has been demonstrated in the infection of athymic BALB/c^nude/nude^ mice that do not possess mature and functional T cells with *R. akari*. BALB/c^nude/nude^ mice infected with *R. akari* and treated with antibiotics to terminate the infection developed antibodies but were not protected against reinfection with *R. akari* [95]. On the other hand, antibodies produced by athymic mice after immunization with heat-killed *R. akari* protected euthymic BALB/c^nude/+^ mice against challenge with *R. akari,* whereas antibodies from immunized euthymic mice failed to protect BALB/c^nude/nude^ mice against this pathogen [95]. These findings indicate protective effects of T cell-independent antibodies and suggest a minor role of T cell-dependent antibodies in primary infection. In another study, however, passive immunization with polyclonal immune serum as well as with monoclonal antibodies against outer membrane proteins A (OmpA) or B (OmpB), but not lipopolysaccharide (LPS), protected C3H/HeN SCID mice from challenge with *R. conorii* [53]. Moreover, the application of polyclonal immune serum even prolonged the survival of C3H/HeN SCID mice with already established *R. conorii* infection and led to reduced bacterial burden [53], demonstrating protective effects.

The protective function of the antibodies depends on the Fc part because Fab fragments of the antibodies were not protective in this model [53]. Antibodies can mediate protection by different mechanisms: (1) the opsonization of particles for the uptake by phagocytes, (2) binding to the pathogen surface and activation of complement which directly attacks the pathogen, or (3) the inhibition of infection via the blockade of molecules that are involved in binding to surface receptors and bacterial uptake. Opsonization of rickettsial particles seems to be at least one mechanism by which antibodies can contribute to protection. Opsonization of *R. conorii* with polyclonal or monoclonal anti-OmpA and anti-OmpB antibodies resulted in enhanced uptake of the bacteria into endothelial cells (SVEC 4-10) and MΦ-like cells (J774A.1) and inhibited bacterial growth in vitro [96]. Polyclonal antibodies and monoclonal anti-OmpB inhibited the phagosomal escape of *R. conorii*, and killing of the bacteria could be ascribed to the release of NO, ROS and l-tryptophan starvation [96]. In passively immunized mice, intraphagosomal killing of *R. conorii* in MΦ and the accumulation of *R. conorii* antigen in these cells was observed [53].

While antibodies against LPS can be detected already on day 6 after the infection of C3H/HeN mice with *R. conorii* [53], specific antibodies that are generated with the help of CD4^+^ T cells appear relatively late in rickettsial infections. For example, antibodies against OmpA and OmpB become detectable as late as on day 12 when *R. conorii*-infected C3H/HeN mice already have recovered [53], and the same is also true for humans. *R. typhi*-infected patients develop a diagnostic antibody titer as late as 15 days after the onset of symptoms [97], and seroconversion in *R. conorii* and *R. africae* infection occurs even later between day 16 and 25 [98]. Overall, the role of antibodies in protection against rickettsial infections is not that clear. It is assumed that antibodies do not play a major role in defense in primary infection but may contribute to protection in secondary infection.

### Mechanisms of T cell-mediated protection against rickettsial infections

T cells clearly play a dominant role in defense against rickettsial infections. Apart from providing help in antibody production by B cells, the probably more important role of CD4^+^ T cells lies within their capacity to release effector molecules. CD8^+^ T cells, on the other hand, are cytotoxic cells that can directly kill infected cells. In recent years, effector mechanisms that are involved in T cell-mediated protection against rickettsial infections have been studied in more detail. In the following, the current knowledge of the mechanisms of CD8^+^ and CD4^+^ T cell-mediated protection is discussed.

### CD8^+^ T cells

CD8^+^ T cells are activated during rickettsial infections and protective against rickettsial infections without doubt. CD8^+^ T cells from *R. conorii*-infected C3H/HeN and *R. australis*-infected C57BL/6 mice show enhanced cytotoxic activity ex vivo against *R. conorii*-infected ECs or J774A.1 MΦ-like cells [46]. The peak response is observed on day 10 post infection [46]. Similarly, CD8^+^ T cells from *R. typhi*-infected C57BL/6 [47] and BALB/c mice [45] express enhanced levels of Granzyme B, demonstrating cytotoxic properties, and release IFNγ. Both Granzyme B and IFNγ expression peak on day 7 post infection in both strains of mice [45, 47]. Interestingly, in *R. typhi*-infected C57BL/6 mice a low level of activated CD8^+^ T cells persists [47], and in BALB/c mice even periodic reactivation of CD8^+^ T cells occurs [45]. As it has been recently shown that *R. typhi* persists in both mouse strains [33], a long-lasting CD8^+^ T cell response as well as CD4^+^ T cell response seems to be important for the control of persisting bacteria. In C3H/HeN mice CD8^+^ T cells are essential for the protection against rickettsial infections. Depletion of CD8^+^ T cells leads to enhanced susceptibility of C3H/HeN mice to *R. conorii* [46, 99] as well as to *R. typhi* [49]. In the absence of CD8^+^ T cells, infected C3H/HeN mice show enhanced bacterial burden and pathology. Furthermore, C57BL/6 MHCI^−/−^ mice that lack CD8^+^ T cells are higly susceptible to a lethal outcome in the infection with *R. australis* compared to wild-type mice [46]. Finally, adoptive transfer of CD8^+^ T cells from immune mice protects C3H/HeN mice against challenge with a normally lethal dose of *R. conorii* [99] and C57BL/6 RAG1^−/−^ mice against lethal infection with *R. typhi,* even when transferred into mice with already established infection [47]. In *R. typhi*-infected C57BL/6 RAG1^−/−^ mice, adoptively transferred CD8^+^ T cells enter the brain which is the organ with the highest bacterial burden in these animals and quickly eliminate the bacteria. Moreover, the mice do not develop clinical symptoms [47]. Similarly, CB17 SCID mice substituted with CD8^+^ T cells are resistant to the infection with *R. typhi* and remain asymptomatic [45]. In contrast to the infection with *R. australis*, however, CD8^+^ T cells are sufficient but not essential for protection in the infection with *R. typhi*. Both C57BL/6 MHCI^−/−^ as well as C57BL/6 MHCII^−/−^ mice, that either lack CD8^+^ or CD4^+^ T cells are equally resistant to *R. typhi*, do not show signs of disease and survive the infection [47].

Because rickettsiae are intracellular pathogens, especially the cytotoxic activity of CD8^+^ T cells has long been considered the most important mechanism of defense. C57BL/6 Perforin^−/−^ mice that lack the cytotoxic potential show a higher susceptibility and lethality upon infection with *R. australis* compared to C57BL/6 IFNγ^−/−^ mice, although being much less susceptible than C57BL/6 MHCI^−/−^ that lack CD8^+^ T cells at all [46]. This indicates a higher contribution of cytoxicity to recovery compared to the release of IFNγ. In fact, the release of IFNγ by CD8^+^ T cells was dispensable for defense against this infection because immune CD8^+^ IFNγ^−/−^ T cells adoptively transferred into *R. australis*-infected C57BL/6 IFNγ^−/−^ mice reduced the bacterial load and conferred protection [46].

In contrast to the infection of mice on the C57BL/6 background with *R. australis*, BALB/c Perforin^−/−^ mice and BALB/c IFNγ^−/−^ mice are as resistant to the infection with *R. typhi* as wild-type mice [45]. Moreover, CB17 SCID mice infected with this pathogen and substituted with either CD8^+^ from BALB/c IFNγ^−/−^ or BALB/c Perforin^−/−^ mice survived the infection without showing symptoms of disease at any point in time [45]. Thus, the cytotoxic activity of CD8^+^ T cells is not essential for protection in the infection with *R. typhi*. In the study mentioned above, *R. typhi*-infected CD8^+^ T cell recipient mice were followed for more than 120 days and then analyzed for the presence of persisting bacteria. Surprisingly, the bacteria were not detectable at all in the organs of CD8^+^ Perforin^−/−^ T cell recipient mice, while CB17 SCID mice that received CD8^+^ T cells from IFNγ^−/−^ mice had bacteria predominantly in the brain and to a much lesser extent in the spleen, lung and liver. None of the animals, however, showed signs of disease [45]. These results indicate that CD8^+^ T cell-derived IFNγ may be even more important than the cytotoxic activity in defense against *R. typhi*, at least in the control of persisting bacteria. They also indicate that persisting *R. typhi* may reside in the brain which is an immune privileged organ.

Overall, it has become clear that the cytotoxic activity as well as the release of IFNγ by CD8^+^ T cells is important for protection against rickettsial infections. In case of *R. typhi* both either the cytotoxic activity or the release of IFNγ by CD8^+^ T cells is sufficient for protection while cytotoxicity seems to play a major role in the infection with other rickettsiae such as *R. australis*.

### CD4^+^ T cells

The enhanced susceptibility of C3H/HeN mice to the infection with *R. conorii* compared to C57BL/6 mice has been connected to the reduced ability of DCs to induce IFNγ-producing CD4^+^ T cells. Bone marrow-derived DCs from C3H/HeN mice that were infected with *R. conorii* showed lower expression of MHCII, reduced release of IL-12p40 and a lower capacity to induce IFNγ release by CD4^+^ T cells than DCs from C57BL/6 mice in vitro [100]. In vivo C3H/HeN mice develop higher frequencies of FoxP3^+^ regulatory T cells than C57BL/6 mice upon *R. conorii* infection which is discussed as a consequence of suppressed CD4^+^ T cell response in these mice [100]. Adoptive transfer of immune CD4^+^ T cells, however, protects C3H/HeN mice against lethal infection with *R. conorii* [99].

Meanwhile, there is increasing evidence that CD4^+^ T cells are sufficient for protection, at least against some rickettsiae. This is clearly true for the infection of mice with *R. typhi*. C57BL/6 MHCI^−/−^ that lack CD8^+^ T cells do not develop disease and survive the infection with *R. typhi* [47] which is in contrast to the infection with *R. australis* where CD8^+^ T cells obviously play a dominant role in defense [46]. Even more important, immune CD4^+^ T cells protect C57BL/6 RAG1^−/−^ mice against lethal challenge with *R. typhi* [47]. Here, CD4^+^ T cells still protect a large percentage of animals with already established infection. Although CD8^+^ T cells are obviously more efficient and quicker in bacterial elimination in this system, CD4^+^ T cells manage to control the bacteria. *R. typhi* was not detectable anymore even in those mice that succumbed to the infection despite CD4^+^ T cell transfer [47]. Similar to CD8^+^ T cells, CD4^+^ T cells enter the CNS of the animals where they induce the expression of iNOS in infiltrating MΦ that harbor *R. typhi* in these mice [33] as well as in microglia which is not observed in CD8^+^ T cell recipient mice [47]. Thus, CD4^+^ T cells enhance the bactericidal activity of these cells in vivo. Activation of microglia and MΦ by CD4^+^ T cells, however, can have pathological effects. *R. typhi*-infected C57BL/6 RAG1^−/−^ that succumbed to the infection despite CD4^+^ T cell transfer still showed enhanced activation of MΦ and microglia in the CNS despite bacterial elimination [47]. In these cases immunopathology seems to be the reason of death.

CD4^+^ T cells express IFNγ in the infection of C57BL/6 as well as of BALB/c mice with *R. typhi* [45, 47]. Generally, the CD4^+^ T cell response in these mice appears with similar kinetics as the CD8^+^ T cell response. Activated CD4^+^ T cells peak on day 7 post infection but do not return to basal levels again, and sporadic reactivation of CD4^+^ T cells is observed in *R. typhi*-infected BALB/c mice [45, 47]. Upon antigen-specific restimulation in vitro CD4^+^ T cells from *R. typhi*-infected BALB/c mice release very high amounts of IFNγ in addition to lower amounts of TNFα. Both of these cytokines have been shown to play an important role in defense against *R. conorii.* The neutralization of either IFNγ or TNFα leads to enhanced pathology in *R. conorii*-infected C3H/HeN mice, and IFNγ-deficient C57BL/6 mice succumb to the infection with a normally sublethal dose of *R. conorii* [101]. Both cytokines likely also play a role in protection against *R. typhi* by CD4^+^ T cells. Immune CD4^+^ T cells from either C57BL/6 or BALB/c mice induce the release of NO from *R. typhi*-infected MΦ and inhibit bacterial growth in vitro. This effect is partially inhibited by the neutralization of IFNγ or TNFα [45, 47]. CD4^+^ T cells adoptively transferred into *R. typhi*-infected CB17 SCID mice protect approximately 90% of the animals from death and provide long-term control of the bacteria [45]. CD4^+^ IFNγ^−/−^ T cells, however, still reduce the growth of *R. typhi* in infected MΦ in vitro. Moreover, CD4^+^ IFNγ^−/−^ T cells are able to eliminate the bacteria and confer protection against *R. typhi* in vivo. 30–90% of *R typhi*-infected CB17 SCID mice that receive these cells survive the infection although showing prolonged disease compared to mice that are substituted with wild-type CD4^+^ T cells [45]. The authors further show that CD4^+^ T cells develop into T_H_17 cells in the infection with *R. typhi* in the absence of IFNγ. Instead of IFNγ, CD4^+^ T cells from *R. typhi*-infected BALB/c IFNγ^−/−^ mice release large amounts of IL-17A and IL-22 and low amounts of IL-17F. These cytokines are not produced at all by immune CD4^+^ T cells from *R. typhi*-infected wild-type BALB/c mice [45], whereas TNFα is released at comparable amounts by both wild-type CD4^+^ and CD4^+^ IFNγ^−/−^ T cells [45]. Other cytokines such as IL-21 that could be involved in bacterial defense are not detectable. Although T_H_17 cells are thought to be mainly involved in defense against extracellular pathogens, cytokines that are produced by these cells can directly contribute to protection against intracellular pathogens. IL-17A/F induce the expression of inflammatory cyokines such as TNFα, IL-1β and IL-6 in tissue cells and MΦ [102–106] and of antimicrobial peptides in tissue cells in concert with IL-22 [107, 108]. Moreover, IL-17A has been shown to promote the expression of iNOS and the release of NO by MΦ infected with *Mycobacterium bovis* bacillus Calmette-Guerin (BCG) [109] and to inhibit the growth of intracellular parasites such as *Chlamydia muridarum* in lung epithelial cells and MΦ in vitro in an iNOS-dependent manner [110] and of *Trypanosoma cruzi* in infected MΦ in vitro which is mediated via the activation of the NAPDH oxidase [111]. This enzyme produces superoxide and other ROS that mediate killing of intracellular pathogens in MΦ and neutrophils similar to NO [112]. Also IL-22 can directly induce microbicidal activity. The growth of *Eimeria falciformis* in infected epithelial cells is inhibited by the treatment with either IFNγ, IL-17A or IL-22 [113]. Furthermore, IL-17A/F induce the release of granulopoetic factors (G-CSF, GM-CSF, SCF) and several chemokines (CXCL-1, CXCL-2, CXCL-5, CXCL-8) that lead to the recruitment of neutrophils that can further contribute to pathogen elimination [114]. Similar mechanisms may be true for defense against *R. typhi* by T_H_17 cells.

Apart from that, IL-17A/F and TNFα can also have pathological effects, which is clearly true in the infection with *R. typhi*. The neutralization of either TNFα or IL-17A leads to much milder disease and enhanced survival of *R. typhi*-infected CB17 SCID mice that received CD4^+^ IFNγ^−/−^ T_H_17 cells [45]. These observations suggest that TNFα and IL-17A exert synergistic pathologcial effects in *R. typhi*-infected mice as described for other inflammatory conditions [115, 116], while combined release of IL-22 and TNFα or IL-22 and IL-17A is beneficial and sufficient for protection. Whether T_H_17 cells play a role in protection and/or pathology in human infection with *R. typhi* is unknown, but one may imagine that these cells may be generated in patients with defective induction of T_H_1 cells, leading to more severe disease. Interestingly, patients with scrub typhus show higher levels of IL-17 than healthy individuals [117]. Moreover, IL-17 levels in patients with headache are higher than in patients without headache, and it is discussed whether IL-17 may be involved in pathophysiology in the infection with *O. tsutsugamushi*, although a direct correlation has not been demonstrated yet [117].

**Fig. 2:**
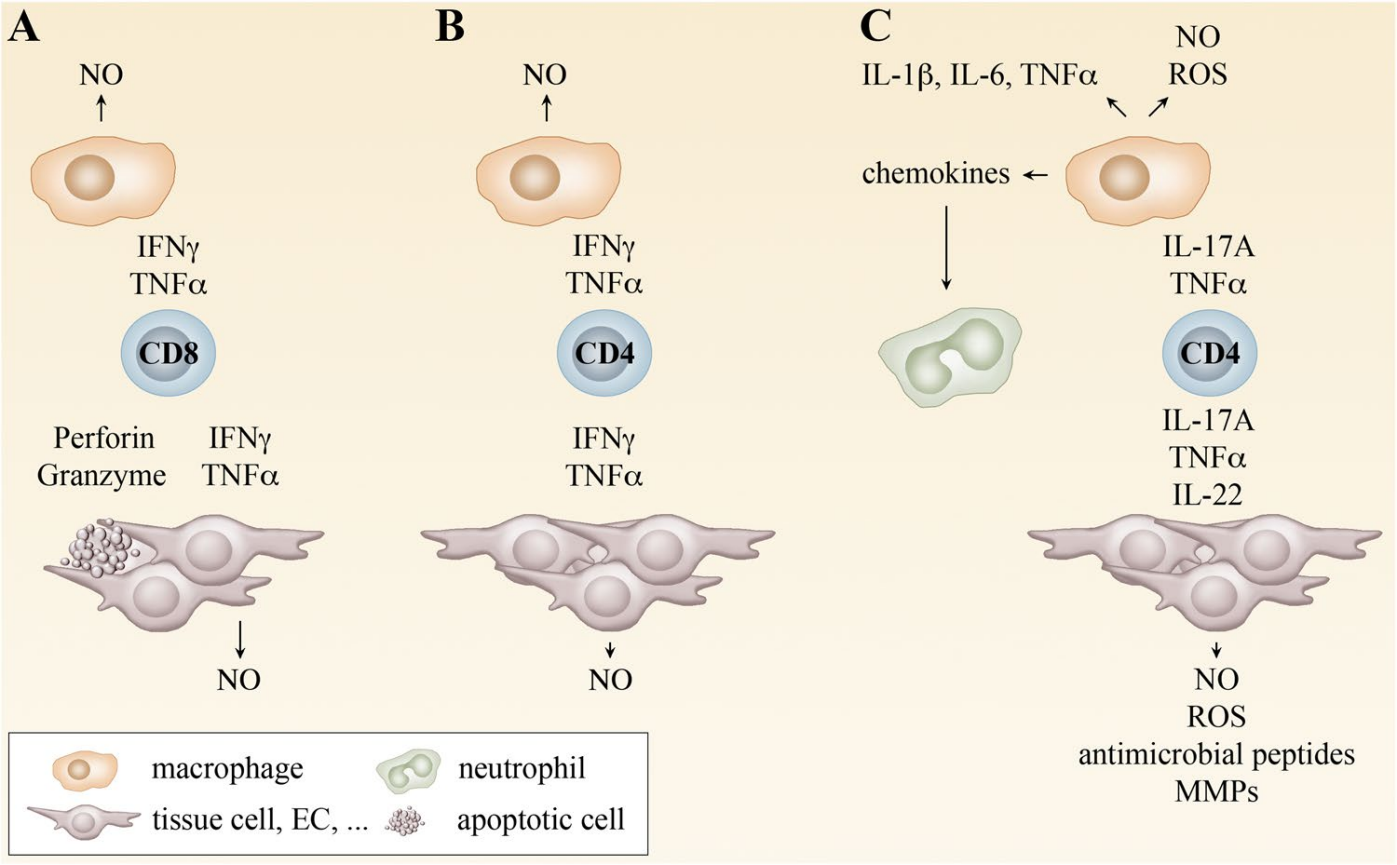
Mechanisms of T cell-mediated protection. CD8^+^ T cells differentiate in the infection with rickettsiae to cytotoxic T cells that induce apoptosis in infected cells via the release of perforin and granzymes. In addition, CD8^+^ T cells produce IFNγ and TNFα. These cytokines induce the production of NO by MΦ and other cells and, thus, enable bacterial killing. The release of cytokines by CD8^+^ T cells is sufficient for protection at least in the infection with some rickettsial species such as *R. typhi* where the cytotoxic activity is not essential for bacterial defense (**a**). CD4^+^ T cells usually differentiate into T_H_1 cells that produce IFNγ and TNFα in the infection with rickettsiae. T_H_1 cells are protective by the induction of NO and bacterial killing by MΦ and other cells (**b**). In the absence of IFNγ, CD4^+^ T cells develop into T_H_17 cells in the infection with rickettsiae. These cells release IL-17A, IL-22 and TNFα. IL-17A and TNFα synergistically induce the production of NO und ROS by MΦ and other cells. In addition, these cytokines induce the release of proinflammatory cytokines. IL-17A further induces the production of chemokines, leading to the recruitment of neutrophils that contribute to local inflammation. IL-22 does not act on immune cells but various tissue cells. IL-22 induces the release of antimicrobial peptides and other factors and can contribute in this way to bacterial elimination. At least in the infection with *R. typhi* T_H_17 cells can be protective. However, the combined release of TNFα and IL-17A is non-beneficial and exerts pathological effects. Inhibition of either TNFα or IL-17A in this situation renders T_H_17 cells as protective as T_H_1 cells (**c**)

The current hypothesis on the mode of action of CD4^+^ T cells is that CD4^+^ T cell-derived cytokines, either IFNγ and TNFα in case of T_H_1 cells or IL-17A and TNFα in case of T_H_17 cells, directly act on MΦ and also neutrophils to activate the bactericidal activity of these cells. IL-22 may contribute to protection by the induction of antimicrobial peptides and other factors in infected non-immune cells such as fibroblasts and endothelial cells. Figure 2 provides an overview on the hypothetic action of CD8^+^ T cells, T_H_1 and T_H_17 cells in defense against rickettsiae. The exact mechanisms how T_H_17 cells mediate protection and/or contribute to pathology in rickettsial infections, however, remain to be elucidated.

### T cell-mediated cross-protection and T cell antigens

Cross-protective immune response between different rickettsial species has been described for animals as well as for humans. Guinea pigs experimentally infected with *R. typhi* are immune to *R. prowazekii* infection and vice versa, and similar solid cross-immunity exists for humans [118]. Spleen cells from *R. conorii*-infected C3H/HeJ mice react to antigen preparations from *R. rickettsii*, *R. sibirica* and *R. australis,* but only poorly to *R. akari*, while spleen cells from *R. akari*-immunized C3H/HeJ mice react to *R. conorii* and other SFG rickettsiae [119]. Moreover, C3H/HeN mice that are infected with a sublethal dose of either *R. conorii* or *R. australis* are protected against a normally lethal challenge with the heterologous pathogen [120]. The same is also true for *R. conorii*- and *R. typhi*-infected C3H/HeN mice [121], demonstrating cross-protection even between SFG and TG rickettsiae and SFG and transitional rickettsiae. Therefore, the identification of immunodominant T cell antigens that are present in a broader range of rickettsiae may lead to the identification of vaccine candidates that can confer protection even against various rickettsiae.

Very recently, five protective *R. prowazekii* CD8^+^ T cell antigens (RP884, RP778, RP739, RP598 and RP403) have been discovered [122, 123]. C3H/HeN mice that were immunized with antigen-presenting cells (APCs) expressing these antigens developed CD8^+^ T cells that expressed IFNγ and Granzym B. Moreover, the animals were protected against a lethal challenge with *R. typhi* [122, 123]. APCs expressing a mix of these antigens were further used for the immunization of C3H/HeN mice followed by the infection with *R. conorii* and conferred at least partial protection [123]. CD8^+^ T cell epitopes have also been identified in the OmpB protein of *R. conorii*. Three synthetic peptides of OmpB (SKGVNVDTV (OmpB_708–716_), ANSTLQIGG (OmpB_789–797_) and IVEFVNTGP (OmpB_812–820_)) induced proliferation, cytotoxic activity, and IFNγ expression by CD8^+^ T cells from *R. conorii*-infected C3H/HeN mice [124]. These proteins and peptides can, therefore, be considered as vaccine candidates. The induction of CD8^+^ T cells, however, is difficult because antigens must be provided to the MHC class I presentation pathway within target cells, which requires cytosolic expression of the respective protein.

Recent findings suggest that CD4^+^ T cells can be as protective as CD8^+^ T cells. Therefore, the induction of CD4^+^ T cells by vaccination may be sufficient for protection against rickettsial infections. CD4^+^ T cells are much easier to induce than CD8^+^ T cells by the application of recombinant protein antigen, leading to MHC class II peptide presentation. So far, only the 22-kDa scrub typhus antigen (sta22) was identified as an antigen against which antibodies as well as CD4^+^ T cells are generated in *O. tsutsugamushi*-infected mice [125]. CD4^+^ T cell antigens from other rickettsial species are still unknown.

## Concluding remarks

Murine models of rickettsial infections have significantly advanced the understanding of immune response against these bacteria. Importantly, not only CD8^+^ T cells but also CD4^+^ T cells can mediate protection. With regard to vaccination, current knowledge suggests that the induction of specific CD4^+^ T cells is sufficient for protection, at least against some rickettsial species. Therefore, CD4^+^ T cell antigens should be identified for the development of a vaccine that induces IFNγ-producing T_H_1 cells. These cells will likely exert less side effects than T_H_17 cells. Such vaccine may even confer cross-protectivity between different rickettsial species.
